# Blockade of Serotonin 5-HT_2A_ Receptors Suppresses Behavioral Sensitization and Naloxone-Precipitated Withdrawal Symptoms in Morphine-Treated Mice

**DOI:** 10.3389/fphar.2016.00514

**Published:** 2016-12-26

**Authors:** Gang Pang, Xian Wu, Xinrong Tao, Ruoying Mao, Xueke Liu, Yong-Mei Zhang, Guangwu Li, Robert W. Stackman, Liuyi Dong, Gongliang Zhang

**Affiliations:** ^1^College of Basic Medical Sciences, Anhui Medical UniversityHefei, China; ^2^College of Medicine, Anhui University of Science and TechnologyHuainan, China; ^3^Jiangsu Province Key Laboratory of Anesthesiology, College of Anesthesiology, Xuzhou Medical UniversityXuzhou, China; ^4^Department of Psychology, Jupiter Life Science Initiative, Florida Atlantic University, JupiterFL, USA

**Keywords:** serotonin, 5-HT_2A_ receptor, morphine, behavioral sensitization, withdrawal, prefrontal cortex, hippocampus

## Abstract

The increasing prescription of opioids is fueling an epidemic of addiction and overdose deaths. Morphine is a highly addictive drug characterized by a high relapse rate – even after a long period of abstinence. Serotonin (5-HT) neurotransmission participates in the development of morphine dependence, as well as the expression of morphine withdrawal. In this study, we examined the effect of blockade of 5-HT_2A_ receptors (5-HT_2A_Rs) on morphine-induced behavioral sensitization and withdrawal in male mice. 5-HT_2A_R antagonist MDL 11,939 (0.5 mg/kg, i.p.) suppressed acute morphine (5.0 mg/kg, s.c.)-induced increase in locomotor activity. Mice received morphine (10 mg/kg, s.c.) twice a day for 3 days and then drug treatment was suspended for 5 days. On day 9, a challenge dose of morphine (10 mg/kg) was administered to induce the expression of behavioral sensitization. MDL 11,939 (0.5 mg/kg, i.p.) pretreatment suppressed the expression of morphine-induced behavioral sensitization. Another cohort of mice received increasing doses of morphine over a 7-day period to induce morphine-dependence. MDL 11,939 (0.5 mg/kg, i.p.) prevented naloxone-precipitated withdrawal in morphine-dependent mice on day 7. Moreover, chronic morphine treatment increased 5-HT_2A_R protein level and decreased the phosphorylation of extracellular signal-regulated kinases in the prefrontal cortex. Together, these results by the first time demonstrate that 5-HT_2A_Rs modulate opioid dependence and blockade of 5-HT_2A_R may represent a novel strategy for the treatment of morphine use disorders.

**Highlights:**

(i) Blockade of 5-HT_2A_ receptors suppresses the expression of morphine-induced behavioral sensitization.
(ii) Blockade of 5-HT_2A_ receptors suppresses naloxone-precipitated withdrawal in morphine-treated mice.
(iii) Chronic morphine exposure induces an increase in 5-HT_2A_ receptor protein level and a decrease in ERK protein phosphorylation in prefrontal cortex.

## Introduction

Morphine and other prescription opioids are the mainstay clinical treatment for moderate to severe acute pain. The increasing prescription of opioids is fueling an epidemic of addiction and overdose deaths (Volkow et al., 2014). In addition, there has been an alarming rise in the illegal use of opioids in the United States over the past two decades (Varrassi and Muller-Schwefe, 2012). Morphine is a highly addictive opioid and the rate of relapse is remarkably high – even after long periods of abstinence (Blondell et al., 2013). The neural mechanisms underlying morphine addiction and relapse remain elusive (Volkow et al., 2014).

Serotonergic fibers originate from raphe nuclei and distribute across the brain, including the prefrontal cortex (PFC) and ventral tegmental area (VTA). Besides its well-documented regulation of sensory perception, mood and cognition, serotonin (5-HT) neurotransmission in the brain affects the development of morphine dependence and the expression of morphine withdrawal (el-Kadi and Sharif, 1995; Muller and Homberg, 2015). There are seven 5-HT receptor families (5-HT_1-7_), encompassing 14 receptor subtypes together with a variety of splice variants (Hannon and Hoyer, 2008). 5-HT_2A_ receptor (5-HT_2A_R) is a G protein-coupled receptor (GPCR) of the type A family. 5-HT_2A_Rs are widely distributed across the central nervous system. 5-HT_2A_Rs regulate cellular function via G protein-dependent, ligand-dependent and ligand-independent signaling pathways, including phospholipase signaling, extracellular signal-regulated kinases (ERK) pathway and tyrosine kinase pathway in neural tissues (Millan et al., 2008; Masson et al., 2012). Pharmacological blockade of 5-HT_2A_Rs presents antipsychotic and antidepressant properties, however, receptor activation possesses cognition enhancement and hallucinogenic properties (Zhang and Stackman, 2015). Accumulating evidence indicates that the 5-HT_2A_R is involved in the behavioral changes elicited by psychostimulants and drugs of abuse. 5-HT_2A_R antagonist M100907 suppressed behavioral hyperactivity induced by cocaine (Fletcher et al., 2002), MK-801, amphetamine (O’Neill et al., 1999) and morphine (Auclair et al., 2004). In addition, M100907 attenuated cocaine priming or cocaine associated cue induced reinstatement of drug-seeking behaviors in rats (Fletcher et al., 2002; Nic Dhonnchadha et al., 2009), and inhibited nicotine priming or nicotine-associated cue induced reinstatement (Fletcher et al., 2012) and nicotine sensitization (Zaniewska et al., 2010). To date, there is little if any reports of the modulation of 5-HT_2A_Rs on morphine-induced behavioral changes.

Chronic opioid treatment can induce a behavioral sensitization, a key factor critical for the acquisition and maintenance of compulsive drug-seeking behavior. The behavioral sensitization may reflect the neuroplasticity associated with addictive drug exposure that causes hyperactivity of neurons that innervate the NAc, such as dopaminergic neurons in VTA, glutamatergic neurons in PFC and basolateral amygdala (BLA; Kalivas and Stewart, 1991). Physical dependence or withdrawal also play a critical role in the reinstatement of drug-seeking behaviors (Robinson and Berridge, 1993). In the present study we investigated the effect of 5-HT_2A_R blockade with MDL 11,939, a potent and selective antagonist for 5-HT_2A_R (Ki values are 0.54 and 2.5 nM at rabbit 5-HT_2A_R and human 5-HT_2A_R, respectively), on morphine-induced behavioral sensitization and withdrawal symptoms and further explored the effect of chronic morphine treatment on 5-HT_2A_R protein level and ERK phosphorylation in mice. Glemanserin (MDL 11,939) has been investigated clinically for the treatment of generalized anxiety disorder (Sramek et al., 1995). Our data revealed that MDL 11,939 suppressed morphine-induced increase in locomotor activity, behavioral sensitization and withdrawal symptoms in male mice.

## Materials and Methods

### Animals

Adult male Kunming mice at 22 ± 2 gram came from the Experimental Animal Center of Anhui Medical University (Hefei, China) and housed in groups of four per cage with *ad libitum* access to food and water. Mouse cages were maintained in a temperature and humidity controlled vivarium with 12/12 h light/dark cycle (lights on at 7:00 am) for at least 1 week prior to experiments. All procedures conducted were in accordance with the guidelines in the National Institutes of Health Guide for the Care and Use of Laboratory Animals and were approved by the Institutional Animal Care and Use Committee at Anhui Medical University.

### Chemicals and Reagents

Morphine hydrochloride was purchased from The First Pharmaceutical Co. Ltd of Shenyang (Shenyang, China), naloxone hydrochloride was obtained from Kangze Pharmaceutical Co. Ltd (Hunan, China), and MDL 11,939 was purchased from Tocris Bioscience (Minneapolis, MN, USA). MDL 11,939 was dissolved in sterile saline containing 1% DMSO, and all other compounds were dissolved in 0.9% sterile normal saline. The drug concentrations were adjusted to an injection volume of 10 ml/kg body weight. The 5-HT_2A_R primary antibody was purchased from Abcam (ab16028, Cambridge, MA, USA). Primary antibodies to (phospho-) ERK1/2 came from Bioworld Technology Inc. (Nanjing, China). The secondary HRP-conjugated anti-mouse IgG antibody for the Western blot (WB) assay came from Beijing Zhong-Shan Biotechnology Co., Ltd (Beijing, China). Enhanced Bicinchoninic acid (BCA) protein assay kit came from Beyotim Institute of Biotechnology (Haimen, China).

### Morphine-Induced Behavioral Sensitization

The morphine-induced behavioral sensitization mouse model was developed based on our previous publication (Zhang et al., 2016b). In brief, mice received morphine (10 mg/kg, s.c.) twice a day (8:00 am and 7:00 pm) for 3 days and then morphine injection was suspended for 5 days. On day 9, a challenge dose of morphine (10 mg/kg, s.c.) was given and the locomotor activity was recorded immediately for 1 h (Li et al., 2008). To examine whether 5-HT_2A_Rs affect the expression of behavioral sensitization, MDL 11,939 (0.5 mg/kg, i.p.) was administered 20 min prior to morphine challenge on day 9 to examine behavioral changes.

### Induction of Morphine Dependence and Withdrawal

The morphine-induced dependence and withdrawal mouse model was developed based on our previous publication (Zhang et al., 2016b). In brief, mice received morphine three times per day starting at a dose of 5 mg/kg (s.c.) and the dose was increased each day until reaching 100 mg/kg by day 6. On day 7, morphine (100 mg/kg, s.c.) was administered at 8:00 am and 2:00 pm. 2.5 h later, the mice received vehicle or MDL 11,939 (0.5 mg/kg, i.p.) treatment followed by naloxone challenge (5.0 mg/kg, i.p.) 15 min later to precipitate morphine withdrawal symptoms. Mice were placed individually into square observation chambers (30 cm × 30 cm × 35 cm tall) and closely observed for 30 min immediately after naloxone challenge.

### Open Field Test and Behavioral Assessment

Horizontal distance traveled and other behavioral phenotypes were tracked with EthoVision XT 5.1 PhenoTyper system (Noldus Information Technology, Wageningen, The Netherlands). The EthoVision software detected the mouse in static subtraction mode (a white mouse on a black background) in each video frame (29.97 fps) and tracked the movement of the digitized pixels that represent the “center point” of the mouse. Distance traveled and immobility were used to assess locomotor activity. A mouse that reared or was grooming would not be detected as immobile (Wu et al., 2015; Zhang et al., 2016b). The withdrawal symptoms quantified included jumping (all feet off the floor), burrowing (escape digging), body grooming, rearing (front feet off the floor), wet dog shakes (whole body shakes), and other behaviors for 30 min (Wu et al., 2015; Zhang et al., 2016b).

### Western Blot

Brains from the mice receiving the escalating morphine treatment were rapidly harvested on day 7 and then stored at -80°C before processing for 5-HT_2A_R WB. The selected brain regions from two mice were pooled together to obtain a sufficient amount of protein necessary for perform WB analysis. Tissue samples obtained from the PFC and hippocampus were homogenized in ice-cold RIPA lysis buffer before centrifuging at 15,000 × *g* at 4°C for 10 min. The supernatant was collected as total proteins and the protein concentration was examined using a BCA protein assay kit. Samples containing 30 μg proteins were separated by 12% SDS-polyacrylamide gel and, then, transferred to polyvinylidene difluoride (PVDF) membranes. After blocking with 5% (w/v) non-fat dry milk and washing in Tris-buffered saline containing 0.1% v/v tween-20 (TBST), the membrane loaded was incubated with the primary antibody (1:100) at 4°C overnight followed by incubation with horseradish peroxidase-conjugated secondary antibody (goat anti-mouse; 1:10,000). Protein bands were visualized by an enhanced chemiluminescent substrate (Thermo Fisher Scientific Inc. Waltham, MA, USA) and captured by the Bioshine ChemiQ4600 imaging system (Shanghai Bioshine Scientific instrument Co., Ltd, Shanghai, China). Densitometry (mean of gray scale) was performed using ImageJ software (NIH). 5-HT_2A_R densitometry values were normalized to corresponding β-actin immunoreactivity in the same lane to reduce any loading and transfer variances among the samples (Zhang et al., 2016b). Each group consists of 3–4 trials.

### Statistical Analysis

Statistical differences in distance traveled and immobility were determined by a two-way repeated measures analysis of variance (ANOVA). If significance was found, a *post hoc* Bonferroni multiple comparison test was used to compare the difference between two groups. Independent samples Student’s *t*-test was used to analyze the difference between two groups. All statistical tests were conducted using the SPSS v19 (IBM, Armonk, NY, USA) software package. Data were expressed as mean ± S.E.M. *P* < 0.05 was considered statistically significant.

## Results

### MDL 11,939 Suppresses Acute Morphine-Induced Hyperactivity

**FIGURE 1:**
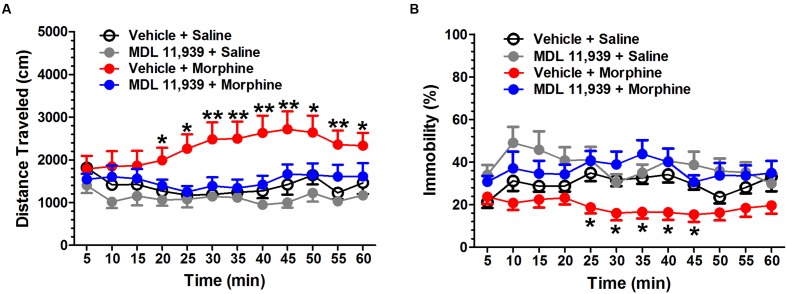
**MDL 11,939 suppresses acute morphine-induced hyperactivity.** MDL 11,939 (0.5 mg/kg, i.p.) was administered 20 min prior to the injection of morphine (5 mg/kg, s.c.). Locomotor activity was recorded for 60 min immediately after morphine administration. **(A)** Morphine-treated mice presented a significant increase in the distance traveled compared with the control mice (vehicle + morphine vs. vehicle + saline, *p* = 0.011). MDL11,939 prevented the morphine-induced increase in locomotor activity (MDL 11,939 + morphine vs. vehicle + saline, *p* = 1.000). Morphine-treated mice presented an increase in the distance traveled from 20 to 60 min compared with the control mice (vehicle + morphine vs. vehicle + saline). **(B)** Morphine treatment significantly suppressed immobility compared with the vehicle (vehicle + morphine vs. vehicle + saline, *p* = 0.047). MDL11,939 prevented the morphine-induced decrease in immobility (MDL 11,939 + morphine vs. vehicle + saline, *p* = 1.000). Morphine-treated mice presented a decrease in immobility from 25 to 45 min compared with the control mice (vehicle + morphine vs. vehicle + saline). Data are expressed as mean ± S.E.M; *n* = 9 in each group; ^∗^*p* < 0.05, ^∗∗^*p* < 0.01 vs. vehicle + saline group.

Systemic MDL 11,939 (0.5 mg/kg, i.p.) did not affect the locomotor activity 20 min after injector (**Supplementary Figure S1**). Therefore, MDL 11,939 was administered 20 min prior to morphine (5 mg/kg, s.c.) injection. The locomotor activity was recorded for 60 min immediately after morphine administration. MDL 11,939 altered morphine-induced increase in locomotor activity. A two-way repeated measures ANOVA on distance traveled measured in 5-min bins revealed a significant main effect of treatment [*F*(3,32) = 6.398, *p* = 0.002] and a significant main effect of treatment × time bin interaction [*F*(33,352) = 2.949, *p* < 0.001], but no effect of time bin [*F*(11,352) = 1.471, *p* = 0.140] (**Figure 1A**). *Post hoc* Bonferroni multiple comparisons showed that morphine-treated mice presented a significant increase in distance traveled compared with the control mice (vehicle + morphine, *n* = 9 vs. vehicle + saline, *n* = 9, *p* = 0.011). MDL 11,939 treatment prevented the morphine-induced increase in locomotor activity (MDL 11,939 + morphine, *n* = 9 vs. vehicle + saline, *p* = 1.000). MDL 11,939 did not alter the distance traveled when compared with the vehicle (MDL11,939 + saline, *n* = 9 vs. vehicle + saline, *p* = 1.000). The distance traveled at each 5-min time bin was further compared with a one-way ANOVA followed by *post hoc* Bonferroni multiple comparisons. Morphine administration increased the distance mice traveled in the task between 20 and 60 min compared with the vehicle (vehicle + morphine vs. vehicle + saline). A two-way repeated measures ANOVA on immobility measured in 5-min bins revealed a significant main effect of treatment [*F*(3,32) = 6.578, *p* = 0.001] and a significant main effect of treatment × time bin interaction [*F*(33,352) = 1.511, *p* = 0.039], but no effect of the time bin [*F*(11,352) = 0.925, *p* = 0.516] (**Figure 1B**). *Post hoc* Bonferroni multiple comparisons showed that morphine-treated mice presented a significant decrease in immobility compared with the control mice (vehicle + morphine vs. vehicle + saline, *p* = 0.047). MDL 11,939 pretreatment prevented the morphine-induced decrease in immobility (MDL 11,939 + morphine vs. vehicle + saline, *p* = 1.000). MDL 11,939 did not alter the distance traveled compared with the vehicle (MDL 11,939 + saline vs. vehicle + saline, *p* = 0.803). The immobility was further compared at each 5-min time bin using a one-way ANOVA followed by *post hoc* Bonferroni multiple comparisons. Morphine administration resulted in a decrease in immobility from 25 to 45 min compared with the control mice (vehicle + morphine vs. vehicle + saline). Moreover, MDL 11,939 at 0.125 and 0.25 mg/kg did not affect the morphine-induced increase in locomotor activity (**Supplementary Figure S2**).

### MDL 11,939 Suppresses the Expression of Morphine-Induced Behavioral Sensitization

**FIGURE 2:**
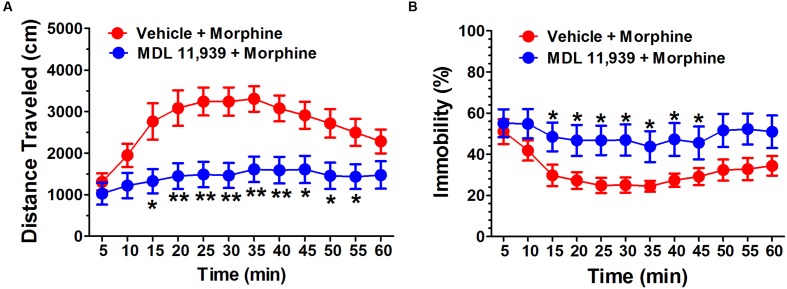
**MDL 11,939 suppresses the expression of morphine-induced behavioral sensitization.** MDL 11,939 (0.5 mg/kg, i.p.) was administered 20 min before the morphine (10 mg/kg, s.c.) challenge on day 9 in morphine behavioral sensitization model. **(A)** MDL 11,939 treatment suppressed morphine-induced increase in distance traveled measured in 5-min time bins compared to the vehicle (*p* = 0.006). **(B)** MDL 11,939 treatment prevented morphine-induced decrease in immobility measured in 5-min time bins compared with the vehicle (*p* = 0.04). Data are expressed as mean ± S.E.M; *n* = 10 in each group; ^∗^*p* < 0.05, ^∗∗^*p* < 0.01 vs. vehicle + morphine group.

MDL 11,939 (0.5 mg/kg, i.p.) was administered 20 min before the morphine (10 mg/kg, s.c.) challenge on day 9 in the morphine behavioral sensitization model. MDL 11,939 altered the morphine-induced behavioral sensitization. A two-way repeated measures ANOVA on distance traveled measured in 5-min time bins revealed a significant main effect of treatment [*F*(1,18) = 9.81, *p* = 0.006], a significant effect of time bin [*F*(11,198) = 19.94, *p* < 0.001] and a significant main effect of treatment × time bin interaction [*F*(11,198) = 4.99, *p* < 0.000] (**Figure 2A**). The distance traveled was further compared at each 5-min bin with an independent samples Student’s *t*-test. MDL 11,939 administration (*n* = 10) suppressed morphine-induced increase in distance traveled during the task from 15 to 55 min compared with the vehicle (*n* = 10). A two-way repeated measures ANOVA on immobility revealed a significant main effect of treatment [*F*(1,18) = 4.89, *p* = 0.04] and a significant main effect of time bin [*F*(11,198) = 7.96, *p* < 0.001], but no effect of the treatment × time bin interaction [*F*(11,198) = 1.51, *p* = 0.131] (**Figure 2B**). The immobility at each time point was compared with an independent samples Student’s *t*-test. MDL 11,939 administration suppressed the morphine-induced decrease in immobility from 15 to 45 min compared with vehicle + morphine treatment.

### MDL 11,939 Suppresses Naloxone-Precipitated Withdrawal in Morphine-Dependent Mice

**FIGURE 3:**
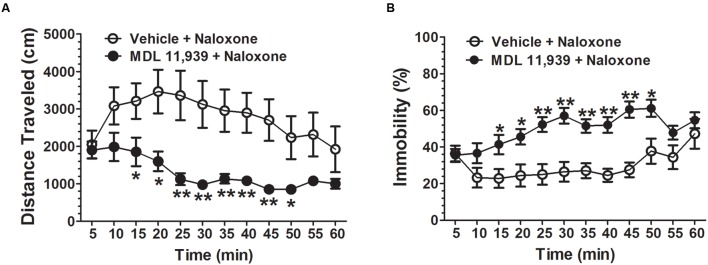
**MDL 11,939 suppresses naloxone-precipitated withdrawal in morphine-dependent mice.** MDL 11,939 (0.5 mg/kg, i.p.) was administered 15 min before the naloxone (5.0 mg/kg, s.c.) challenge to precipitate morphine withdrawal. **(A)** MDL 11,939 treatment suppressed naloxone-precipitated increase in distance traveled measured in 5-min time bin (*p* = 0.004). **(B)** MDL 11,939 treatment suppressed naloxone-precipitated decrease in immobility measured in 5-min time bin (*p* = 0.001). Data are expressed as mean ± S.E.M; *n* = 10 in each group; ^∗^*p* < 0.05, ^∗∗^*p* < 0.01 vs. vehicle + naloxone group.

MDL 11,939 (0.5 mg/kg, i.p.) was administered 15 min prior to the naloxone (5.0 mg/kg, s.c.) challenge in order to precipitate morphine withdrawal. MDL 11,939 altered naloxone-precipitated withdrawal in morphine-treated mice. A two-way repeated measures ANOVA on distance traveled measured in 5-min time bin revealed a significant main effect of treatment [*F*(1,18) = 10.63, *p* = 0.004], a significant main effect of time bin [*F*(11,198) = 6.53, *p* < 0.001] and a significant effect of treatment × time bin interaction [*F*(11,198) = 2.20, *p* = 0.016] (**Figure 3A**). The distance traveled was further compared at each 5-min time bin using an independent samples Student’s *t*-test. MDL 11,939 (*n* = 10) suppressed morphine-induced increases in distance traveled from 15 to 50 min compared with the vehicle + morphine group mice (*n* = 10). A two-way repeated measures ANOVA on immobility measured in 5-min time bin revealed a significant main effect of treatment [*F*(1,18) = 16.79, *p* = 0.001], a significant main effect of time bin [*F*(11,198) = 5.51, *p* < 0.001], and a significant effect of treatment × time bin interaction [*F*(11,198) = 3.46, *p* < 0.001] (**Figure 3B**). The immobility was further compared at each 5-min time bin with an independent samples Student’s *t*-test. Systemic MDL 11,939 suppressed morphine-induced decreases in immobility from 15 to 50 min compared with the vehicle + morphine group. Moreover, MDL 11,939 administration suppressed jumping behavior in morphine withdrawal mice (**Table 1**).

**Table 1:** MDL 11,939 ameliorates naloxone-precipitated withdrawal symptoms in morphine-dependent mice.

Group	*n*	Jumping	Burrowing	Body grooming	Rearing	Scratch	Wet dog shakes	Head shakes	Digging body	Paw licking	Face grooming	Penile grooming	Extended posture
Vehicle	10	23.10 ± 2.35	12.90 ± 2.35	9.20 ± 1.68	3.80 ± 1.03	16.60 ± 2.59	2.80 ± 0.56	2.60 ± 0.56	14.70 ± 1.77	11.40 ± 2.10	12.30 ± 2.22	3.60 ± 0.86	3.89 ± 0.59
MDL 11,939	10	11.30 ± 1.23^∗∗^	8.80 ± 1.31	8.10 ± 1.22	3.20 ± 2.22	13.20 ± 1.93	1.80 ± 0.55	2.90 ± 0.43	8.00 ± 1.70^∗^	7.10 ± 1.48	8.20 ± 1.01	1.70 ± 0.47	4.22 ± 0.57
*t*(18)		4.44	1.53	0.53	0.25	-0.41	1.28	-0.42	2.73	1.67	1.68	1.94	1.05
*p*		<0.001	0.145	0.603	0.81	0.69	0.22	0.68	0.014	0.11	0.11	0.68	0.31


*Mice received progressively increasing doses of morphine for 7 days. 2.5 h after the last injection, mice received MDL 11,939 (0.5 mg/kg, i.p.) 15 min before naloxone challenge. The behavioral performance was recorded for 30 min immediately after naloxone challenge. Data are expressed as mean ± S.E.M.; ^∗^*p* < 0.05; ^∗∗^*p* < 0.01 vs. vehicle.*

### Chronic Morphine Treatment Is Associated with an Increase in 5-HT_2A_R Protein Level and a Decrease in ERK Phosphorylation in PFC and Hippocampus

**FIGURE 4:**
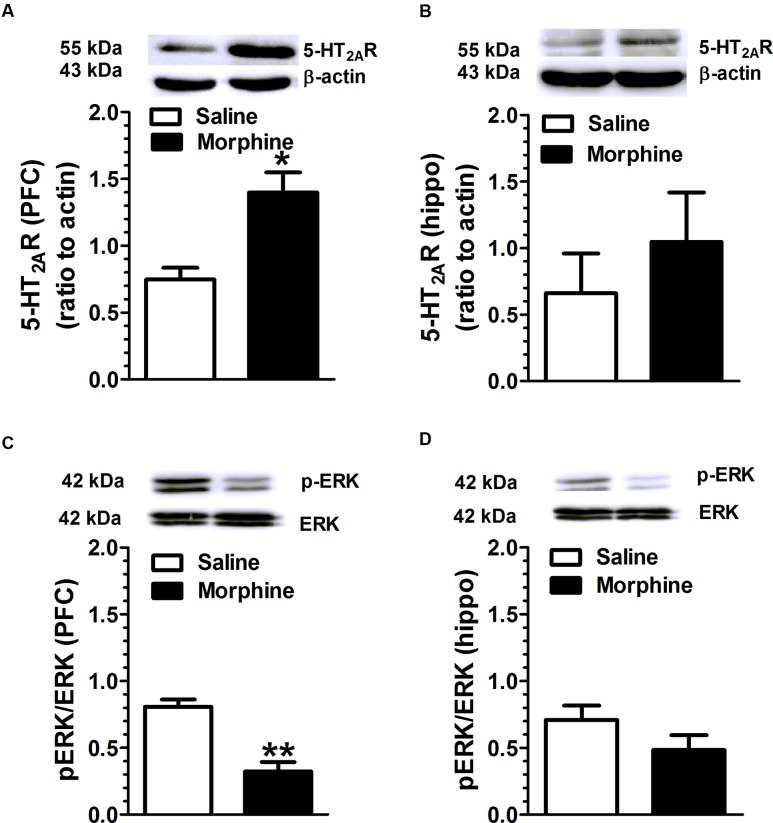
**Chronic morphine treatment is associated with an increase in 5-HT_2A_R expression and a decrease in ERK phosphorylation in the PFC and hippocampus.** Mice received increasing doses of morphine for 7 days to develop morphine dependence, and brain was harvested 2.5 h after last injection. **(A)** Chronic morphine treatment was associated with an increase in 5-HT_2A_R expression in PFC (*p* = 0.02), and **(C)** a decrease in the ratio of pERK/ERK in PFC (*p* = 0.001). The hippocampus also presented a trend of an increase in 5-HT_2A_R expression **(B)** and a decrease in ERK phosphorylation **(D)**. Data are expressed as mean ± S.E.M; *n* = 10 in each group; ^∗^*p* < 0.05, ^∗∗^*p* < 0.01 vs. saline group.

Mice received increasing doses of morphine over 7 days to develop morphine dependence, and brain tissue was harvested 2.5 h after last morphine injection. 5-HT_2A_R, pERK and ERK levels in PFC and hippocampus were detected using WB assay. Chronic morphine treatment was associated with an increase in 5-HT_2C_R expression [*t*(4) = 3.75, *p* = 0.02], and a decrease in the ratio of pERK/ERK in PFC [*t*(6) = 5.43, *p* = 0.001] (**Figure 4**) compared with the vehicle treatment. Similarly, morphine treatment induced a trend of increase in 5-HT_2A_R expression and a trend of decrease in ERK phosphorylation in hippocampus.

## Discussion

The present studies examined the modulation of 5-HT_2A_Rs on morphine-induced increases in locomotor activity, behavioral sensitization, and withdrawal in mice. Our results indicated that blockade of 5-HT_2A_Rs with MDL 11,939 suppressed morphine-induced hyperactivity, behavioral sensitization, and naloxone-precipitated withdrawal symptoms. Moreover, repeated morphine treatment was associated with an increase in 5-HT_2A_R expression and a decrease in ERK phosphorylation in PFC. Together, these results by the first time demonstrates that 5-HT_2A_Rs modulate morphine dependence and pharmacological blockade of 5-HT_2A_Rs may be a novel strategy for the treatment of opioid use disorders.

Our results confirmed that a systemic blockade of 5-HT_2A_Rs suppressed morphine-induced increase in locomotor activity. Previous studies revealed that acute and chronic morphine treatments increased locomotor activity, DA and 5-HT release, which could be prevented by the 5-HT_2A_R antagonist SR46349B pretreatment (Tao and Auerbach, 1995; Auclair et al., 2004). Morphine suppress the neuronal firing of VTA GABAergic interneurons via μ-opioid receptors, leading to a disinhibition of VTA dopaminergic neurons and an increase in DA release (Johnson and North, 1992). It suggests that morphine induces locomotor hyperactivity by suppressing GABAergic neurons and, subsequently, the resultant boosting of dopaminergic neurotransmission (Iwamoto, 1981). A subpopulation of VTA dopaminergic neurons expresses 5-HT_2A_R (Doherty and Pickel, 2000). Local infusion of 5-HT_2A_R antagonist M100907 in VTA prevented cocaine induced hyperactivity (McMahon et al., 2001). These indicate that MDL 11,939 attenuates the 5-HT_2A_R-mediated excitatory effect on VTA dopaminergic neurons, and suppresses morphine-induced locomotor hyperactivity.

Behavioral sensitization is developed by drug-induced immediate molecular and/or cellular changes in the VTA. DA neurotransmission within the NAc plays a vital role in the expression of behavioral sensitization (Kalivas and Stewart, 1991). Our results revealed that blockade of 5-HT_2A_Rs inhibited the expression of behavioral sensitization in morphine-treated mice. It suggests that MDL 11,939 attenuates the 5-HT_2A_R-mediated excitatory effect on NAc dopaminergic neurons, and suppresses morphine-induced behavioral sensitivity. Consistently, 5-HT_2A_R antagonist SR46349B prevents the development and expression of morphine-induced sensitization (Auclair et al., 2004).

Physical dependence and withdrawal symptoms are characteristics of morphine addiction. The phenotypes of morphine withdrawal in rodents include jumping, ptosis, diarrhea, wet dog shakes, and motivational dysfunction (Zhang et al., 2016b). It is assumed that locus coeruleus (LC) modulates jumping, rearing, and locomotor activity, periaqueductal gray matter regulates rearing and locomotor activity, and anterior preoptic hypothalamus and nucleus raphe magnus controls wet dog shake (Koob et al., 1992). Morphine exposure suppresses LC neuronal activity, while abrupt morphine termination disinhibits LC neurons and increase LC neuronal firing, leading to withdrawal symptom precipitation (Taylor et al., 1988). Blockade of 5-HT_2A_Rs attenuated the neuronal excitation critical for precipitating morphine withdrawal. The mechanisms behind need further investigation.

Results from WB assay revealed that chronic morphine treatment increased 5-HT_2A_R protein expression and decreased in ERK phosphorylation in PFC. The PFC is a critical brain region associated with drug addictive behaviors. For example, Chemical lesions of the mPFC disrupt the development of cocaine and amphetamine-induced sensitization (Wolf et al., 1995), suggesting that increase in 5-HT_2A_R expression is a critical neuroadaptative component resulted from morphine exposure. The signaling and functional significances of the morphine-induced 5-HT_2A_R protein overexpression remains illusive. Identify the subcellular distribution of 5-HT_2A_Rs, receptor signaling and functional characteristics will be necessary to understand the protein change. Interestingly, ERK phosphorylation was decreased in both the PFC and hippocampus to chronic morphine treatment. Although activation of 5-HT_2A_Rs can increase pERK1/2 formation (Millan et al., 2008; Masson et al., 2012), ERK phosphorylation is regulated by multiple cellular signals, and the inconsistency between 5-HT_2A_R expression and pERK accumulation may be attributed to other factors.

MDL 11,939 is a potent and selective 5-HT_2A_R antagonist. Glemanserin (MDL 11,939) was clinically investigated for the treatment of generalized anxiety disorder due to its efficacy, safety, as well as the probability to cross the blood-brain barrier (Sramek et al., 1995). In the present study, we used MDL 11,939 as a tool to explore the neurobiological mechanism of morphine-induced behavioral change. Based on our data, MDL 11,939 may serve as a medical treatment for morphine use disorders in human. 5-HT_2A_Rs are expressed predominately in nociceptive sensory neurons in lumbar dorsal root ganglia and participate in pain regulation (Van Steenwinckel et al., 2009). MDL 11,939 has been show to suppress 2′,3′-dideoxycytidine-induced neuropathic pain in rats; 5-HT_2A_R knock-out mice did not develop 2′,3′-dideoxycytidine-induced neuropathy (Van Steenwinckel et al., 2008). We previously demonstrated that MDL 11,939 could suppress acetic acid-induced acute pain, incision pain and sciatic nerve ligation neuropathic pain in mice (unpublished). The influence of MDL 11,939 on anxiety and pain may participate in its effects on morphine-induced behavioral changes.

Drug exposure is associated with oxidative stress in the brain (Guzman et al., 2006; Abdel-Zaher et al., 2013; Schiavone et al., 2016). Chronic morphine treatment induces a progressive increase in brain glutamate and oxidant malondialdehyde (MDA) levels and a progressive decrease in brain non-enzymatic antioxidant, intracellular glutathione (GSH) levels and enzymatic antioxidant, GSH-Px activity (Abdel-Zaher et al., 2013). Nitric oxide synthase inhibitors, phospholipase A2 inhibitors and free radical scavengers attenuate the expression of naloxone-precipitated withdrawal syndrome in morphine-dependent mice (Mori et al., 2007; Abdel-Zaher et al., 2013). The Nicotinamide Adenine Dinucleotide Phosphate (NADPH) oxidase NOX enzyme is a major contributor of oxidative damage in the CNS, being involved in a variety of brain disorders. Mice with NOX1 mutation present an augment of morphine-induced analgesia and an inhibition of tolerance (Ibi et al., 2011). Serotonergic neurotransmission may also participate in the morphine-induced oxidative stress. Morphine increases 5-HT release and oxidative metabolism (Enrico et al., 1998). The 5-HT metabolite melatonin can also scavenge free radicals and abort oxidative stress present in damaged tissue (Ebadi et al., 1998). Glutamate can induce oxidative stress (Atlante et al., 2001). We previously demonstrated that activation of 5-HT_2A_R enhances glutamate release (Zhang et al., 2016a). It is postulated that MDL 11,939 attenuates oxidative stress by blockade of 5-HT_2A_Rs and subsequent glutamate release. It is interesting to further investigate serotonergic neurotransmission in morphine-induced oxidative stress and addiction.

The present studies revealed that blockade of 5-HT_2A_R suppresses the expression of morphine-induced behavioral sensitization and ameliorates naloxone-precipitated withdrawal symptoms. In addition, chronic morphine exposure increase 5-HT_2A_R protein level within the PFC. 5-HT_2A_R may be a potential target for the treatment of morphine use disorders.

## Author Contributions

GZ conceived and designed this experiment and wrote the manuscript. GP, XW, and GL conducted experiment and data analysis. XT, Y-MZ, LD, and RS interpreted the data and wrote the manuscript. RM and XL conducted the supplementary experiment.

## Conflict of Interest Statement

The authors declare that the research was conducted in the absence of any commercial or financial relationships that could be construed as a potential conflict of interest.
