# Supplementary material for: Blockade of Serotonin 5-HT2A Receptors Suppresses Behavioral Sensitization and Naloxone-Precipitated Withdrawal Symptoms in Morphine-Treated Mice
Source: Front Pharmacol. 2016 Dec 26;7:514. doi: 10.3389/fphar.2016.00514 (PMC5183621; doi:10.3389/fphar.2016.00514)
Supplement: Supplementary file 1 [file Data_Sheet_1.DOCX]

**Supplementary information**

**Effects of MDL 11,939 (0.5 mg/kg, i.p.) on distance traveled and immobility in drug-naïve mice**

Mice were administered with MDL 11,939 (0.5 mg/kg, i.p.; n = 8) or vehicle (n = 9). The locomotor activity was recorded immediately for 45 min. MDL 11,939 altered the locomotor activity. A two-way repeated measures ANOVA on distance traveled measured in 5-min time bin revealed a significant main effect of time (*F*(8, 120) = 5.645, *p* < 0.001), but no effects on the treatment (*F*(1, 15) = 4.366, *p* = 0.054) and time × treatment interaction (*F*(8, 120) = 0.752, *p* = 0.646). The effect of MDL 11,939 on distance traveled was further compared at each 5-min time bin. MDL 11,939 suppressed distance traveled at 5 and 15 min (*p* < 0.05). A two-way repeated measures ANOVA on immobility measured in 5-min time bins did not reveal significant effects of time (*F*(8, 120) = 1.649, *p* = 0.118), treatment (*F*(1,15) = 2.985, *p* = 0.105) and time × treatment interaction (*F*(8, 120) = 0.531, *p* = 0.831).


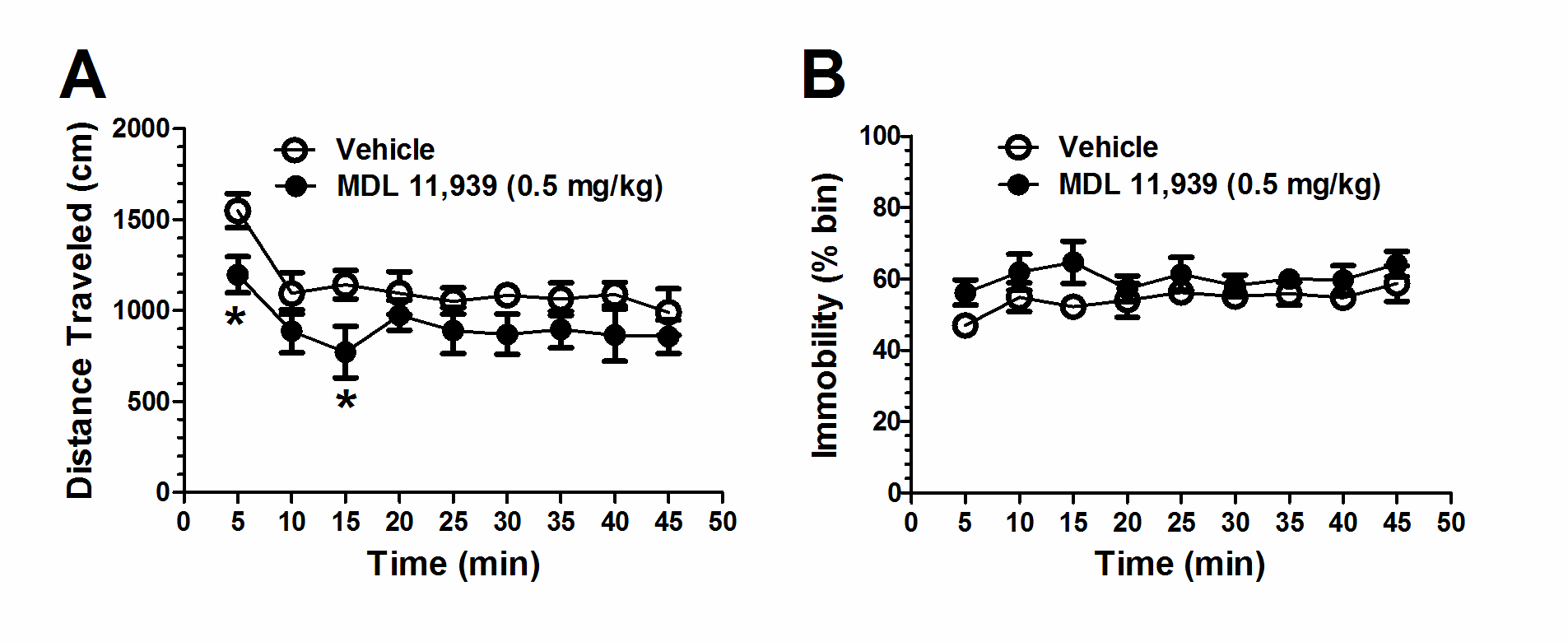


**Figure S1**. Effects of MDL 11,939 (0.5 mg/kg, i.p.) on distance traveled and immobility in mice. (A) MDL 11,939 suppressed the distance traveled at 5 and 15 min after drug treatment. (B) MDL 11,939 did not affect the immobility. Data are expressed as mean ± S.E.M; n = 8-9 in each group; **p* < 0.05 vs. vehicle group.

**MDL 11,939 (0.5 mg/kg, i.p.) suppresses morphine-induced increase in locomotor activity**

Mice were administered with MDL 11,939 (0, 0.125, 0.25 and 0.5 mg/kg, i.p.; n = 8-11) 20 min before morphine (5.0 mg/kg) challenge. The locomotor activity was recorded immediately for 60 min. A one-way ANOVA on distance traveled revealed a significant main difference (*F*(3, 32) = 3.48, *p* = 0.027). Post hoc Bonferroni multiple comparisons showed that MDL 11,939 at 0.5 mg/kg significantly reduced the distance traveled compared to the control mice. There was no difference in immobility (*F*(3, 32) = 1.03, *p* = 0.393).


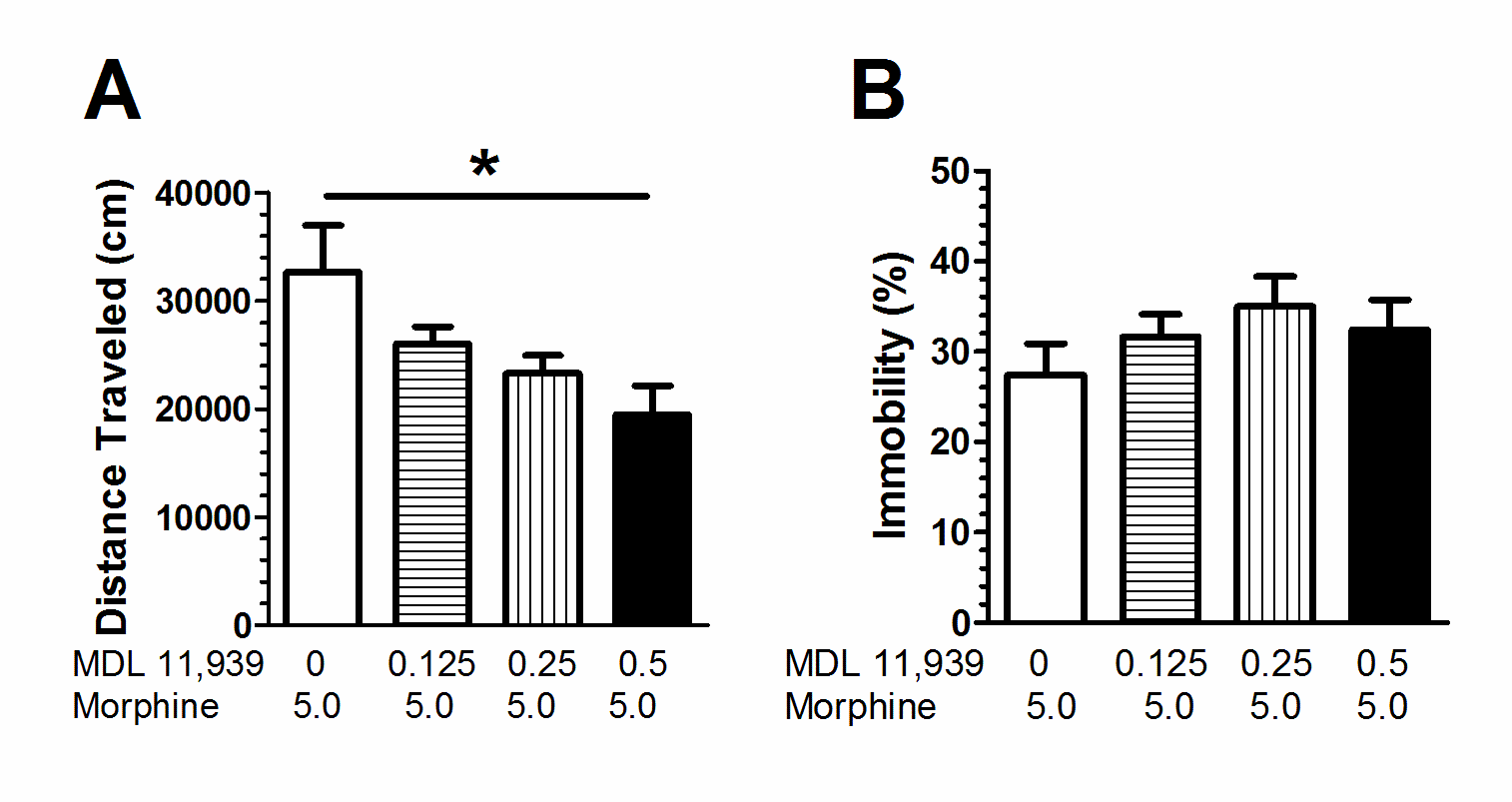


**Figure S2**. Effects of MDL 11,939 on distance traveled and immobility in mice. Mice received MDL 11,939 (0, 0.125, 0.25, or 0.5 mg/kg, i.p.) 20 min before morphine (5.0 mg/kg, s.c.) treatment. (A) MDL 11,939 at 0.5 mg/kg suppressed the distance traveled. (B) MDL 11,939 did not affect the immobility. Data are expressed as mean ± S.E.M; n = 8-11 in each group; **p* < 0.05 vs. vehicle group.
